# High-Efficiency Production of Large-Size Few-Layer Graphene Platelets via Pulsed Discharge of Graphite Strips

**DOI:** 10.3390/nano9121785

**Published:** 2019-12-16

**Authors:** Xin Gao, Tomomasa Hiraoka, Shunsuke Ohmagari, Shigeru Tanaka, Zemin Sheng, Kaiyuan Liu, Meng Xu, Pengwan Chen, Kazuyuki Hokamoto

**Affiliations:** 1 State Key Laboratory of Explosion Science and Technology, Beijing Institute of Technology, Beijing 100081, China; rich.c.gao0522@163.com; 2 Institute of Pulsed Power Science, Kumamoto University, Kumamoto 860-8555, Japan; 176d8552@st.kumamoto-u.ac.jp (T.H.); 198d8454@st.kumamoto-u.ac.jp (S.O.); tanaka@mech.kumamoto-u.ac.jp (S.T.); 3 School of Material Science and Engineering, Beijing Institute of Technology, Beijing 100081, China; zmshengbit@163.com (Z.S.); 13121164743@163.com (K.L.); xumeng@bit.edu.cn (M.X.)

**Keywords:** graphene, large size, pulsed wire discharge, graphite strip

## Abstract

The synthesis of large-size graphene materials is still a central focus of research into additional potential applications in various areas. In this study, large-size graphene platelets were successfully produced by pulsed discharge of loose graphite strips with a dimension of 2 mm × 0.5 mm × 80 mm in distilled water. The graphite strips were made by pressing and cutting well-oriented expanded graphite paper. The recovered samples were characterized by various techniques, including TEM, SEM, optical microscopy (OM), atomic force microscopy (AFM), XRD and Raman spectroscopy. Highly crystalline graphene platelets with a lateral dimension of 100–200 μm were identified. The high yield of recovered graphene platelets is in a range of 90–95%. The results also indicate that increasing charging voltage improves the yield of graphene platelets and decreases the number of graphitic layers in produced graphene platelets. The formation mechanism of graphene platelets was discussed. This study provides a one-step cost-effective route to prepare highly crystalline graphene platelets with a sub-millimeter lateral size.

## 1. Introduction

Graphene, firstly synthesized by Noveselov et al. [1] in 2004, has been proved as a wonderful future material with plenty of applications [2,3,4,5,6,7,8]. As a typical two-dimensional material, its particular atom-thick graphitic layer structure consisting of carbon atoms packed into a honeycomb lattice possesses various unique properties compared with other carbon nanomaterials [9], including outstanding thermal transport property [10,11], superconductivity [12,13], mechanical properties [5,6,14], optical properties [15,16], electronic properties [1,4,17], etc. A great deal of research has suggested multiple applications of graphene in areas such as field effect transistors [1,18], sensors [19,20], metal-free electrodes [21,22], energy storage [23], biomedical applications [24], etc. In addition, the applications of graphene materials also depend on their specific morphology [4,23], lateral dimension [25,26,27], and quality [25,27]. In much of the research conducted on graphene, one central focus is to synthesize large-size graphene materials for a higher potential application in the areas of electronics, solar energy devices, and mechanical structures [28,29,30]. For example, Ruse et al. [25] demonstrated that graphene with a large size increases the hydrogen storage of Mg/graphene nanocomposite by bridging Mg nanoparticles. Xu et al. [29] also employed the graphene at a size of 50 μm–1 mm grown on Ni film substrate to improve the light transmittance in the mid-infrared (MIR) bandwidth of HgCdTe infrared detector with low cost. Additionally, Zhang et al. [31] synthesized large area uniform graphene film consisting of 1–30 μm graphene platelets to replace indium tin oxide (ITO) as flexible, transparent conductive film.

Various methods have been reported to synthesize graphene materials [9,32,33,34,35,36] including mechanical exfoliation method, oxidation reduction method, chemical vapor deposition (CVD) method, liquid-phase exfoliation method, arc-discharge method, electrochemical exfoliation method, detonation method, pulsed wire discharge method, interlayer catalytic exfoliation, etc. Among them, several methods, including oxidation reduction method, CVD method, and interlayer catalytic exfoliation, have been reported to prepare large-size graphene. Su et al. [37] obtained ultra large single-layer reduced graphene oxide (up to millimeter size) using oxidation reduction method (Hummer’s method). In this study, natural graphite flakes with an average size of 3–5 mm were oxidized by a mixture of concentrated H_2_SO_4_, P_2_O_5_ and K_2_S_2_O_8_, exfoliated by stirring with addition of KMnO_4_, and then reduced using hydrazine monohydrate to form large-size graphene. Through CVD method, the wafer-scale graphene was also synthesized [38] on different metal substrates, using the deposition of carbon atoms from CH_4_ or C_2_H_4_ gases at high temperatures (800–1077 °C). Bae et al. [39] synthesized 30-inch graphene films using the same method. With respect to interlayer catalytic exfoliation method, Geng et al. [28] employed FeCl_3_-intercalated graphite mixed with 30% H_2_O_2_ in a reactive bottle for 2 h to exfoliate the graphitic layers to form graphene in a size of 40 × 60 μm^2^ with scalable production. However, till now, a great challenge has remained in realizing large-quantity and high-quality production of large-size graphene materials for further industrialization.

Pulsed wire discharge (electrical wire explosion) employs a high-density current to heat a thin conductive wire, leading to rapid melt and vaporization of the wire at high temperature and pressure [40]. Subsequently, the ultra-hot products scatter out rapidly along a shockwave, and then cool down in the medium to form nanoparticles. Thus, the whole process of pulsed wire discharge, whose duration is in nano- or micro-seconds, can be divided into two processes, joule heating process and subsequent explosion process [35]. The wire types, media and the energy input adjusted by charging voltage are the main factors influencing the joule heating process of pulsed wire discharge [35,40,41,42], providing a one-step cost-effective route to synthesize nanoparticles. Various nanomaterials have been synthesized through this method, including metal nanopowders, metal compound nanopowders, nanocomposite materials, and multiple carbon nanomaterials. For example, Liu et al. [41] reported the synthesis of Al nanoparticles by pulsed discharge of Al wire in an Argon medium. Wada et al. [42,43] obtained TiO_2_ and TiN nanopowders using the pulsed discharge of Ti wire in air and liquid nitrogen, respectively. Tanaka et al. [44] have recovered tungsten carbide nanoparticles after the pulsed discharge to tungsten wire in liquid paraffin. He et al. [45] synthesized boron nitride nanosheet/nanotube-Fe nanocomposite via pulsed discharge of iron wire coated by boron nitride powder. Furthermore, several metal/metal oxide/graphene nanocomposites were also prepared through pulsed discharge of different metal wires in graphene oxide suspension [46,47]. In addition, several reports have been documented to use pulsed wire discharge to synthesize carbon nanomaterials, including fullerene [48,49], carbon nanotube [50,51], and amorphous carbon [52]. However, the synthesis of graphene materials with a sub-millimeter lateral size using pulsed wire discharge has rarely been reported.

In our previous study, a graphite stick was connected to electrodes for pulsed discharge to prepare mono-layer and few-layer graphene in distilled water [35]. Through delicate control of energy input, the graphitic layers in as-prepared graphene materials can be generally controlled. The lateral size of recovered graphene materials was 0.5–5 μm, determined by the initial size of graphite particles in the raw graphite stick. In this study, we demonstrate a one-step route with high yield to prepare few-layer graphene platelets (FLGP) with a lateral dimension of 100–200 μm through pulsed discharge of thin graphite strips made of well-oriented expanded graphite paper.

## 2. Materials and Methods

### 2.1. Sample Prepararion

**Figure 1:**
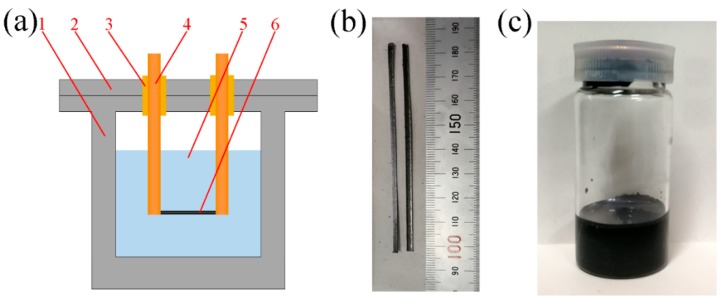
(**a**) Schematic diagram of pulsed wire chamber, 1—cylindrical stainless steel container, 2—lid, 3—insulator, 4—copper electrodes, 5—distilled water, 6—graphite wire, photographs of (**b**) graphite strip, and (**c**) recovered suspension of No. 3 sample.

To recover the as-prepared samples, a cylindrical stainless steel explosion chamber (Figure 1a) with an inner diameter of 200 mm and a depth of 300 mm was designed. The chamber consists of a lid and a cylindrical container. Two copper electrodes wrapped with insulation blocks were installed with the lid and connected to a capacitor with a capacitance of 12.5 μF. The capacitor connected to electrodes could be charged by a power supply to certain voltages (0–40 kV) to control the stored energy. The discharge process was controlled by an air switch in the discharge circuit. Expanded graphite paper was pressed and cut to obtain graphite strips with a dimension of 2 mm × 0.5 mm × 80 mm and a mass of 0.113 g (Figure 1b). The graphite strip was fixed to the two copper electrodes for pulsed discharge. Then 5 L distilled water was poured into the chamber to immerse the graphite strip. After pulsed discharge, the black aqueous mixture (Figure 1c) was collected in a glass bottle, then dried to remove all the water for further characterization.

### 2.2. Characterizations

The morphology and microstructure of as-prepared samples were examined by optical microscopy (OM, Nikon LM 2), and analyzed by field emission scanning electron microscopy (SEM, JEOL JSM 6510A) and high resolution transmission electron microscopy (HRTEM, FEI Tecnai G^2^ F20 S-Twin). Raman spectra of the raw graphite strip and recovered samples were recorded on a LabRAM Aramis Raman spectrometer with a He-Ne laser at an excitation wavelength of 633 nm. The corresponding XRD patterns were recorded on an X-ray diffractometer (XRD) (Rigaku Ultima IV multipurpose XRD system) with Cu Kα radiation (λ = 0.15406 nm) at a step size of 0.05° (2θ) and a step scan time of 1.0 s. The sample suspensions were dropped on a Si plate and dried at room temperature for optical microscopic, SEM and Raman spectroscopic analysis. The sample suspensions were also dropped and dried on a copper mesh grid for TEM analysis. The atomic force microscopy (AFM) analysis was taken by MFP-3D Infinity AFM.

## 3. Results

The experimental conditions of pulsed discharge of graphite strips are listed in Table 1, including the charging voltage and the energy stored in the capacitor. Three charging voltages (20, 30, and 40 kV) were selected for pulsed discharge experiments. Moreover, the graphene yields for these experiments are calculated by measuring the mass of the recovered graphene and initial graphite strip. The yield results are listed in Table 1.

**Figure 2:**
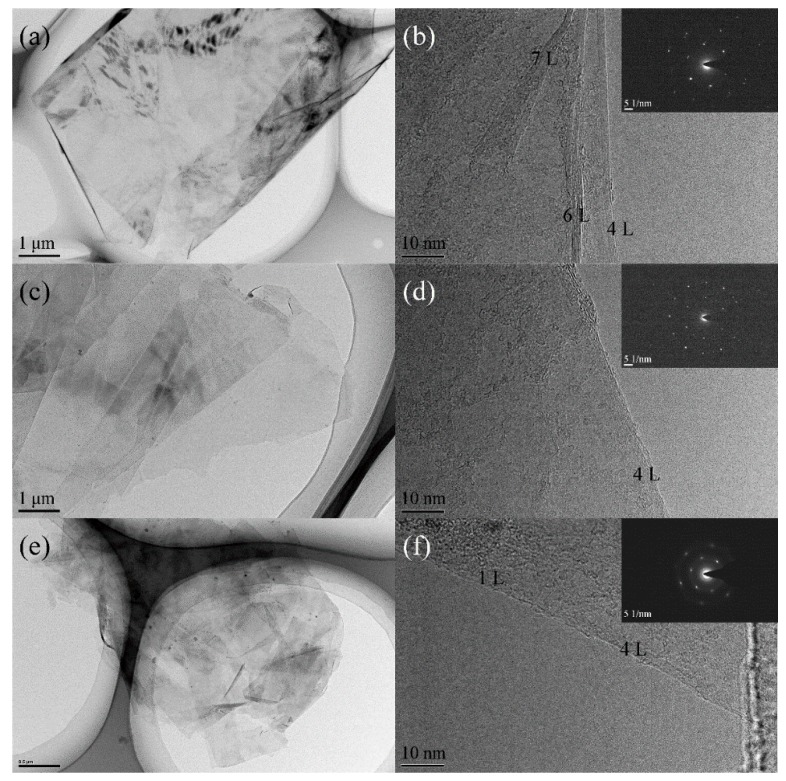
(**a**) TEM and (**b**) HRTEM images of No. 1 sample, (**c**) TEM and (**d**) HRTEM images of No. 2 sample, (**e**) TEM and (**f**) HRTEM images of No. 3 sample. The insets of (b), (d), and (f) show the selected area electron diffraction (SAED) patterns of Nos. 1–3 samples, respectively.

Figure 2 shows the microstructures of the recovered samples examined by TEM and HRTEM. The results of typical TEM images show the presence of ultra-thin wrinkled and extended carbon films with an interlayer distance of 0.3–0.4 nm. Based on the numbers of graphitic layers in the above films, the recovered samples are identified as a mixture of mono-layer graphene and FLGP. When the charging voltage was 20 kV (No. 1 sample), the recovered sample mainly contained FLGPs with 3–7 graphitic layers. As the charging voltage increased to 30 kV (No. 2 sample), the main component was few-layer graphene consisting of 2–5 graphitic layers. When the charging voltage further increased to 40 kV (No. 3 sample), the recovered sample exhibited a mixture of mono-layer graphene and FLGPs with 2–4 graphitic layers. The results imply that the increase of charging voltage is conducive to produce FLGPs with less layers. Furthermore, the selected area electron diffraction (SAED) patterns of all three samples display hexagonally arranged diffraction spots, which is in accordance with the typical results of highly crystalline graphene.

**Figure 3:**
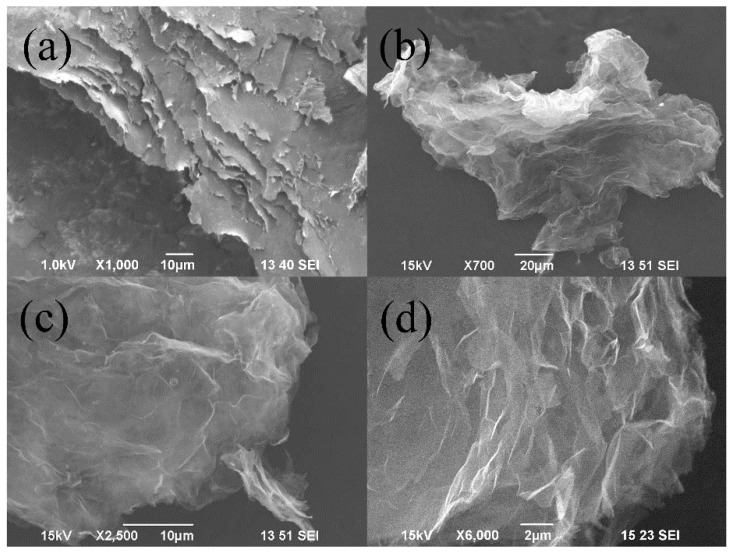
(**a**) SEM image of the cross section of the raw graphite strip, (**b**) SEM, and (**c**), (**d**) higher magnification SEM images of No. 3 sample, respectively.

Figure 3a shows the cross sectional microstructure of raw graphite strips, indicating a layered structure consisting of quantities of graphitic layers. Figure 3b–d show typical SEM and higher-magnification SEM images, respectively, demonstrating the presence of curved and extended ultra-thin carbon films which is the typical morphology of graphene [28,53,54,55]. The curved structure is owing to thermodynamically instability of the two dimensional materials [56]. The statistics of SEM examinations show the size of produced FLGPs is in a range of 100–200 μm, which is much larger than that (0.5–5 μm) of graphene nanosheets prepared by pulsed discharge of graphite sticks.

**Figure 4:**
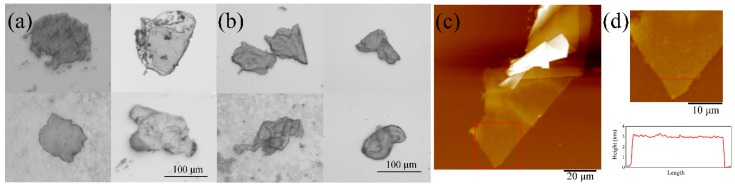
OM images of (**a**) sample No. 1 and (**b**) sample No. 3, (**c**) and, (**d**) AFM topography images of sample No. 1, respectively. (**d**) also shows the height profile in of the few-layer graphene platelets (FLGP) along the red line.

Furthermore, the OM images of samples No. 1 and 3 (Figure 4a,b) show the transparent FLGPs with wrinkles and folds, which is similar to the morphology of the OM results of graphene which were also reported by Escobar-Alarcon et al. [57] and Lee et al. [58]. The statistical results of OM images also show the lateral sizes of produced FLGPs in samples No. 1–3 are in a range of 100–200 μm. It implies that the charging voltage has little influence on the lateral size of produced FLGPs produced by pulsed discharge. In addition, Figure 4c shows the typical AFM topography image of sample No. 1, revealing the presence of ultra-thin carbon films with a size of 100 μm. Figure 4d shows a higher magnification image of the carbon film with the height profile along the red line. The height result is in a range of 2.7–3.1 nm, which most probably corresponds to the thickness of 5–7 layer graphene, considering the thickening phenomenon due to tip–graphene electrostatic force, physisorbed water, etc. [59,60] The thickness and size of FLGP obtained by AFM are in good agreement with those obtained by HRTEM, SEM, and OM.

**Figure 5:**
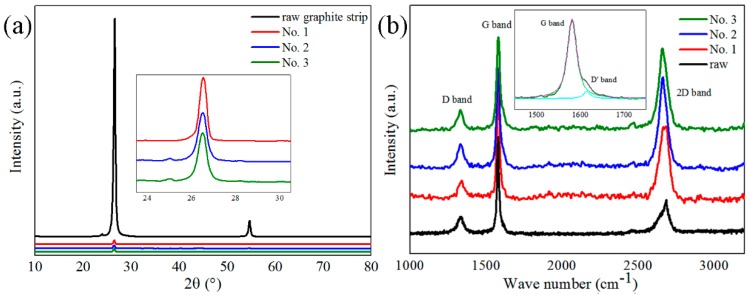
(**a**) XRD patterns and (**b**) Raman spectra of the raw graphite strip and recovered samples.

Figure 5a shows the XRD patterns of the raw graphite strip and the recovered samples. The XRD pattern of raw graphite strip shows only two characteristic peaks, one strong peak appearing at 26.5° assigned to graphite (002) diffraction with a weak peak at 54.5° assigned to graphite (004) diffraction, indicating the interlayer distance of graphitic layers with high crystallinity. The absence of (101) diffraction at 44.4° in the XRD pattern reveals good orientation of raw graphite strips. While in the XRD patterns of recovered samples, only a weak diffraction peak is observed at 26.2–26.4° (inset of Figure 5a), assigned to graphene (002) diffraction, demonstrating the existence of FLGPs produced by pulsed discharge. The calculated d values corresponding to the (002) peaks of recovered samples are in range of 0.337–0.340 nm based on Bragg’s law. The results are slightly larger than the d values of graphite (0.335 nm), also in a good agreement with the TEM results and the previous reports [22,59].

Figure 5b shows the Raman spectra of the raw graphite strip and recovered samples, providing a quick and simple structural and quality characterization of above carbon materials. Four typical Raman bands of carbon materials are observed in Figure 5b, including D band (1332 cm^−1^), G band (1581 cm^−1^), D’ band (1617 cm^−1^, as shown in inset of Figure 5b), and 2D band (in a range of 2662–2688 cm^−1^). The 2D band has been widely used to distinguish mono-layer graphene and few-layer graphene from graphite [60,61]. A higher value of I_2D_/I_G_ indicates less graphitic layers [54,56] in produced FLGPs. The I_D_/I_G_ value is used to characterize the disorder degree of the graphitic layers [61,62]. The I_D_/I_D’_ reveals the disorder type of graphene [63].

The above intensity ratio values of the raw graphite strip and the recovered samples are listed in Table 1. The I_2D_/I_G_ values of the recovered samples were 0.70–1.03, much larger than that of the raw graphite strip (0.29), indicating that the recovered samples were few-layer graphene [54,56]. Moreover, when the charging voltage was increased from 20 kV to 30 kV and 40 kV, the I_2D_/I_G_ value increased from 0.70 to 0.91 and 1.03 accordingly, indicating that increasing the charging voltage can decrease the number of graphitic layers of produced graphene. Additionally, the 2D band position also revealed the thickness of the graphene materials [64]. In Figure 5b, as the charging voltage was increased from 20 kV to 40 kV, a slight redshift of the 2D band from 2673 cm^−1^ to 2662 cm^−1^ was observed, revealing that the graphitic layers in FLGPs are less when the charging voltage is higher. The results of the influence of charging voltage on graphene layers is in good agreement with the TEM results. In addition, all the I_D_/I_G_ values of recovered samples are in a range of 0.15–0.20 similar to that of the raw graphite strip (0.17), suggesting that the disorder degree of the recovered samples and the raw graphite strip are nearly the same. Thus, the pulsed discharge process in our experiments can hardly generate structural defects. Furthermore, the above I_D_/I_G_ values are also smaller than those of graphene produced by arc discharge method (ca. 1.0) [65], oxidation reduction method (ca. 1.0) [37,66,67], and electrochemical exfoliation method (ca. 0.42) [68], showing higher crystallinity of FLGPs produced by pulsed discharge. The I_D_/I_D’_ values of the recovered samples were in the range of 2.88–3.69, close to 3.5, suggesting that the main disorder in the raw material and formed FLGPs is boundary-like disorder [63]. It also indicates that the action of pulsed discharge can barely lead to the vacancy defects, in which the I_D_/I_D’_ is close to 7 [63]. Raman results are in good agreement with those of TEM and XRD analysis.

The yields of produced FLGPs under different conditions are listed in Table 1, demonstrating a high yield of 90–95%, which is much higher than those of many other methods, such as pulsed discharge of graphite sticks (40–50%) [35], arc-discharge method (10–20%) [65], liquid phase exfoliation (7–51%) [69], several mechanical exfoliation methods [33], etc. The yield results also indicate that the increase of charging voltage from 20 kV to 30 kV and 40 kV improves the yield of produced FLGPs from 90% to 92% and 95%, respectively.

## 4. Discussion

**Figure 6:**
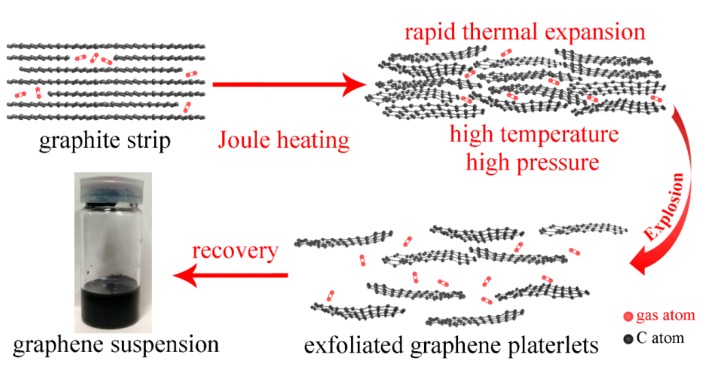
Schematic illustration of formation of FLGPs in pulsed discharge of graphite strips.

Based on the above characterization results, we propose the mechanism regarding the formation of FLGPs using pulsed discharge (as shown in Figure 6) which is similar to those of pulsed discharge of graphite sticks and thermal exfoliation method. During the joule-heating process of pulsed discharge, the rapid energy input leads to an environment of high temperature and high internal pressure. Under the action of high temperature and high internal pressure, the graphitic layers in well-oriented graphite strips overcome the constraints of Van der Waals force and π-π interaction, and are exfoliated easily to form FLGPs. After exfoliation, the formed FLGPs are separated in the distilled water medium due to the rapid expansion during explosion process, which prevents the stacking and agglomeration of formed FLGPs from Van der Waals force. Thus, the FLGP suspension is obtained.

However, note that the graphite strip in these experiments is a well-oriented graphite material, in which the lateral dimension of graphitic layers is in sub-millimeter scale, rather than the graphite stick consisting of micro graphite particles with randomly-distributed orientation. Since the expansion direction of graphitic layers is approximately along the z-axis of graphite crystallites and vertical to the surface of graphite strips, the expansion of well-oriented graphite crystallites can be rarely inhibited by the expansion of adjacent crystallites. Consequently, the exfoliation efficiency of graphite strips is high. Moreover, the density of the graphite strip is approximately 1.4 g/cm^3^, lower than the theoretical density of graphite (2.26 g/cm^3^) and the density of the graphite stick (1.9 g/cm^3^) in Ref 35, implying a loose structure and high porosity of graphite strip. During pulsed discharge, under the sharp increase of temperature and pressure, the gas molecules in this loose material expand rapidly to exfoliate graphitic layers. Thus, both good orientation and the gas in the loose structure are conducive to the exfoliation under high temperature and high internal pressure, leading to a higher exfoliation efficiency of the graphite strip and a higher yield (90–95%) of FLGPs.

The energy input during pulsed discharge is controlled to only cause the rapid thermal expansion effect to exfoliate graphitic layers without destroying the atomic structure of the graphitic layers, therefore the high crystallinity and large dimension of the original graphite crystallites in the graphite strips results in the high crystallinity and large lateral dimension (100–200 μm) of the exfoliated FLGPs after pulsed discharge. In addition, a higher charging voltage increases the energy input during pulsed discharge, resulting in a higher temperature and internal pressure. Consequently, when the charging voltage is increased, the exfoliation efficiency is also increased to generate a slightly higher yield of FLGPs with less graphitic layers.

## 5. Conclusions

This study demonstrates an efficient, cost-effective and environmentally friendly route to produce highly crystalline large-size FLGPs in distilled water at room temperature.

The highly crystalline FLGPs with a lateral dimension of 100–200 μm were produced successfully with a high yield of 90–95%. The mechanism of graphene production is supposed to be the exfoliation of graphitic layers from a graphite strip under the action of joule heating induced by pulsed discharge. Two factors, charging voltage and the loose structure of the graphite strip, are critical to the exfoliation of graphitic layers and the exfoliation efficiency. As the increase of charging voltage from 20 to 40 kV, the yield of FLGPs is slightly increased with less graphitic layers owing to the higher exfoliation efficiency induced by higher internal pressure.

Additionally, the main cost of this experiment is the graphite strips cut from graphite paper with a cost of $22.8 per kilogram (product from Jinglong Special Carbon Co., Ltd., Beijing, China), implying the great economical advantage of the synthetic route compared with the price of commercial graphene (e.g., graphene from Sigma-Aldrich, St. Louis, MO, USA ($1191.1 per kilogram), etc.). Moreover, the experiments were carried out using graphite strips in distilled water without any other chemical agents, indicating that this route is environmentally friendly. Considering the high yield of this route and the large lateral dimension of produced graphene, this route could have a high potential in the areas of electronics, solar energy devices, and mechanical structures.

## Figures and Tables

**Table 1:** Experimental conditions and characterization of pulsed discharge of graphite strips *.

No.	U (kV)	E (J)	2D Band (cm^−1^)	I_2D_/I_G_	I_D_/I_G_	I_D_/I_D’_	Yield
Raw	-	-	2684	0.29	0.17	3.58	-
1	20	2500	2673	0.70	0.15	3.69	90%
2	30	5625	2665	0.91	0.19	2.89	92%
3	40	10,000	2662	1.03	0.20	2.88	95%

* U is the charging voltage, E is the stored energy in the capacitor, I_2D_/I_G_, I_D_/I_G_, and I_D_/I_D’_ are the intensity ratios of 2D band to G band, D band to G band, and D band to D’ band of Raman spectra, respectively.
